# Spatial variability in the amount of forest litter at the local scale in northeastern China: Kriging and cokriging approaches to interpolation

**DOI:** 10.1002/ece3.5934

**Published:** 2019-12-26

**Authors:** Qianqian Qin, Haiyan Wang, Xiangdong Lei, Xiang Li, Yalin Xie, Yonglin Zheng

**Affiliations:** ^1^ College of Forestry Beijing Forestry University Beijing China; ^2^ Institute of Forest Resource Information Techniques Chinese Academy of Forestry Beijing China

**Keywords:** cokriging, kriging, litter amount, principal component analysis

## Abstract

Litter is essential to promote nutrient cycling and maintain the sustainability of forest resources. However, its variability has not been sufficiently studied at the local scale. The prediction of litter amount using ordinary cokriging with Pearson correlation analysis (COK_P_) and ordinary cokriging with principal component analysis (COK_PCA_) was compared with that using ordinary kriging (OK) based on cross‐validation at the local scale of a 1‐ha plot over natural spruce–fir mixed forest in Jilin Province, China. Litter samples in semidecomposed (F) and complete decomposed (H) horizons were collected using an equidistant grid point sampling of 10 m × 10 m. Pearson correlation analysis and principal component analysis (PCA) were used to confirm auxiliary variables. The results showed that the amount of litter was 19.65 t/ha in the F horizon and 10.37 t/ha in the H horizon. The spatial structure variance ratio in the H horizon was smaller than that in the F horizon, indicative of its stronger spatial autocorrelation. Spatial distributions of litter amount in both horizons exhibited a patchy and heterogeneous pattern. Of the selected stand characteristics and litter properties, litter moisture content indicated the strongest relationship with litter amount. Cross‐validation revealed that COK_PCA_ using the comprehensive score as an auxiliary variable produced the most accurate map. The average standard error and root‐mean‐square error between the predicted and measured values were always smaller, the mean error and mean standardized error were much closer to 0, and the root‐mean‐square standardized error was closer to 1 than COK_P_ using litter moisture and OK. Therefore, a clear advantage of cokriging based on principal component analysis was observed and COK_PCA_ was found to be a very useful approach for further interpolation prediction.

## Graphical abstract

We compared three spatial interpolation methods (ordinary kriging, ordinary cokriging with Pearson correlation analysis, and ordinary cokriging with principal component analysis) for better estimating the spatial pattern of litter amount in forest ecosystems. Our results provided evidence that cokriging based on principal component analysis can be successfully applied to improve the prediction of litter amount.

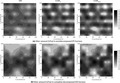

## 1 INTRODUCTION

Litter amount, as a principle resource for improving forest quality, has been found to determine the growth rate of plants, through nutrient return and utilization (Godoy, Castro‐Díez, Logtestijn, Cornelissen, & Valladares, 2010; Maguire, 1994; Miller, 1984; Sayer, 2006; Teramage, Onda, Kato, & Gomi, 2014; Ukonmaanaho, Merilä, Nöjd, & Nieminen, 2008). However, litter amount is often characterized by heterogeneity at different scales around the world, including the local scale (Burnham, 1997; Xia, Chen, Schaefer, & Detto, 2015; Yamashita et al., 2010), landscape scale (Lu & Liu, 2012; Parsons, Congdon, Shoo, Valdez‐Ramirez, & Williams, 2014), and regional and global scales (Bray & Gorham, 1964; Liu et al., 2004; Lonsdale, 1988). Spatial variation refers to the difference and diversity in different spatial locations within a certain region, affecting the distribution pattern of the community or landscape, and it is common but very important feature in forest ecosystems (Burt & Butcher, 2010; Li & Reynolds, 1995; Urban, O'Neill, & Shugart, 1987). Given that it is essential to understand biogeochemical cycles and other ecosystem functions, and because many biological and nonbiological processes that occur at a large scale are rooted in smaller‐scale changes, there is a need to study the spatial heterogeneity of the amount of forest litter at the local scale.

Litter amount is linked to litter input and decomposition, as well as other factors such as topography, microclimate, vegetation composition, geological substrates, and soil physicochemical properties (Aceñolaza, Zamboni, Rodriguez, & Gallardo, 2010; Hermansah, Masunaga, Wakatsuki, & Aflizar, 2002; Kitayama & Aiba, 2002; Proctor, Nderson, Ogden, & Vallack, 1983; Röderstein, Hertel, & Leuschner, 2005; Staelens et al., 2011). There are particular controlling factors that are important in different research areas or at different scales. For example, similar latitude, altitude, slope, temperature, and precipitation have relatively weak effects on the spatial variability of the amount of litter at the local scale, and thus particular attention has been paid to the relationships between litter amount and stand characteristics and the properties of litter itself. Previous studies dealt with the effects of stand characteristics on litter amount and decomposition (Cseresnyés, Csontos, & Bózsing, 2006; Jonard, 2008; Prescott, 2002; Sariyildiz, Anderson, & Kucuk, 2005; Trogisch, He, Hector, & Scherer‐Lorenzen, 2016). These studies suggest that modifying stand conditions could result in complex influences on the amount of litter. Litter amount can affect the rates of decomposition by changing physicochemical characteristics and resource variability in litter horizons (Fang, Zhao, Zhou, Huang, & Liu, 2015; Gripp et al., 2018; Parsons et al., 2014). Conversely, the discrepancies in litter properties at different decomposition stages may also result in variability of litter amount. Recently, Petraglia et al. (2018) reported that litter quality not only shows the strongest relationship with early litter decomposition dynamics, but it also regulates the impact of water availability on mass loss. Owing to all the correlative factors, considerable uncertainties remain in the prediction of spatial variability in litter amount. This is notably true for mixed stands and natural forests, which have higher biodiversity and variability in canopy composition, and a higher rate of localized disturbances (Nickmans, Jonard, Verheyen, & Ponette, 2019; Wang, Wang, & Huang, 2008).

Geostatistics is a typical method that is used for investigating spatial patterns and interpolations based on measured values and distance between detecting points (Liu et al., 2014). Ordinary kriging (OK) is most often used for unbiased optimal interpolation of regionalized variables (Ferguson, Newbury, Maxted, Ford‐Lloyd, & Robertson, 1998). Its capability to account for spatial dependence directly relies on the quantity and quality of the sample data (Miller, Franklin, & Aspinall, 2007), but its predictive ability is limited in regions with complicated environmental factors. Ordinary cokriging (COK) makes use of the correlation between different regionalized variables (Odeh, McBratney, & Chittleborough, 1995) and is an extension of kriging for the optimal estimation of regionalized variables developing from a single attribute to two or more coregionalized attributes (Goovaerts, 1998). The initial comparison between kriging and cokriging was based on theoretical analysis that indicated the superiority of cokriging and that it ensured consistency between an estimation of a sum and the separate estimation of each of its terms (Wackernagel, 1994). Recently, the enhancement of cokriging for spatial interpolation has been investigated in practical application (Adhikary, Muttil, & Yilmaz, 2017; Basaran et al., 2011; Kuntz & Helbich, 2014; Yang, Luo, Jiang, Li, & Yuan, 2016). Basaran et al. (2011) concluded that cokriging was superior to kriging in predicting soil hydraulic conductivity with limited available data on a 12.8 km^2^ area. In their study, the water‐stable aggregate was significantly related to soil hydraulic conductivity, and thus it was used as an auxiliary variable for the estimation of soil hydraulic conductivity. Yang et al. (2016) found that cokriging improved the result by 45.5% compared with kriging, with soil bulk density as a primary variable and soil water content as an auxiliary variable. Moreover, a few studies combined geostatistics with principal component analysis (PCA) in order to extract auxiliary variables (Borůvka, Mládková, Penížek, Drábek, & Vašát, 2007; Levi & Rasmussen, 2014; Markhvida, Ceferino, & Baker, 2018). Borůvka et al. (2007) applied the combination of geostatistics and PCA to study how stand factors affect the spatial distribution of soil characteristics. All these interpolation methods have mainly been applied to the studies of soil properties, but how to assess litter amount using cross‐validation and select comprehensive auxiliary variables is not fully understood.

Our study was based on a 100 m × 100 m permanent plot over natural spruce–fir mixed forest that was established for litter sample collection in the semidecomposed (F) and complete decomposed (H) horizons and the survey of stand characteristics before the peak of fall (late August) in Jilin Province, China. Spatial variability of litter amount was evaluated using geostatistical analysis, and three spatial interpolation methods, including OK, COK_P_ (using the strongest correlation with litter amount as an auxiliary variable), and COK_PCA_ (using the comprehensive score as an auxiliary variable), were compared. The present study attempted to (a) examine spatial variability of litter amount in the F and H horizons at the local scale, (b) test the relationships of litter amount with selected factors of stand characteristics and litter properties, (c) investigate the possibility of using different auxiliary variables to predict litter amount, and (d) compare kriging with cokriging in litter amount estimation at the local scale based on cross‐validation.

## 2 MATERIAL AND METHODS

### 2.1 Study site and sample plot

**Figure 1:**
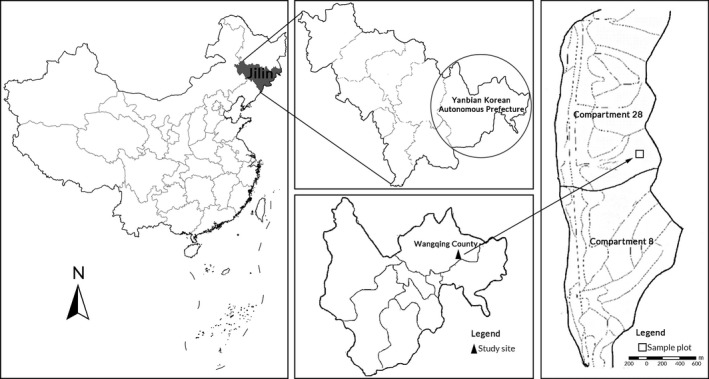
Location of the study area in northeastern China

This study was conducted at the Jingouling Forest Farm, Wangqing County, Jilin Province (43°17′–43°25′N, 130°05′–130°20′E, an altitude of 300–1,200 m, a slope of 5–25°, see Figure 1). The climate is influenced by north temperature monsoon with a mean annual temperature of 3.9°C and an average annual precipitation ranging from 600 to 700 mm. Soils are developed over basalt, gneiss, or granite and are predominantly Bor‐Udic Cambisols (Gong, 1999), which is equivalent to Humic Cambisols (IUSS Working Group WRB, 2014). The forest types that are present include coniferous forest, broad‐leaved forest, and mixed coniferous and broad‐leaved forest. In July 2012, we established a 100 m × 100 m plot (Figure 1) and further divided it into 100 subplots (10 m × 10 m) in natural spruce–fir mixed forest, with a mean altitude of 773 m, mean slope of 3°, and northeastern aspect. The stand density was 1,437 stem/ha, with the main tree species of *Abies nephrolepis*, *Picea jezoensis*, *Pinus koraiensis*, *Picea koraiensis*, *Betula platyphylla, Populus ussuriensis*, *Ulmus pumila*, *Fraxinus mandschurica,* and *Tilia tuan*. The average diameter at breast height (DBH), tree height, and stand volume in the forest was 14.0 cm, 15.1 m, and 209.1 m^3^/ha, respectively.

### 2.2 Sample collection and stand survey

In August 2017, within the center of each 10 m × 10 m subplot, litter was collected in a quadrat of 50 cm × 50 cm, and it was separated into F and H horizons as there was little or even no litter in the undecomposed (L) horizon at this time. The F horizon consists of medium to strongly fragmented material with many mycelia and thin roots, whereas the H horizon is made up of a humified amorphous material (Papamichos, 1990). Subsequently, digital images of the canopy were obtained using Nikon fish‐eye lenses. Tree species and DBH for the individual trees and stem number for each subplot were recorded if DBH was larger than 5 cm.

### 2.3 Determination of litter properties and calculation of stand characteristics

The determination of litter amount, moisture content, organic carbon (OC), total nitrogen (TN) and total phosphorous (TP) concentrations, and calculation of stem number, species number, the proportion of conifer stems and species, Gleason (*D*), Pielous (*J*) and Shannon Wiener (*H*′) indices, and basal area were conducted following descriptions by Qin et al. (2019). In addition, canopy density was analyzed using the digital images of the canopy, which were processed using Adobe Photoshop (version CS6, Adobe Systems Software Ireland Ltd) and calculated as follows (Qi, Luo, & Zhao, 2009):

(1) $$\varepsilon =1-\frac{e}{E}$$

where, *ε* is the canopy density, *e* and *E* are the pixel value of sky portion in the selected area and that of the selected area, respectively. The selected area is encompassed by the fish‐eye lense images that we processed to remove forest land, undergrowth bush and trees outside the plot.

### 2.4 Descriptive statistical analyses

The mean, maximum, minimum, and standard deviation (*SD*) were calculated for the items of each horizon. The Kolmogorov–Smirnov statistical test, together with skewness and kurtosis values, was performed to check whether the data were normally distributed, and if not, logarithm transformation was used (Limpert & Stahel, 2011). Pearson correlation analysis was used to test the correlations of litter amount with affecting factors. The PCA is a statistical analysis method that converts multiple indices into a few principal components (PCs) to reduce dimensionality, and it is calculated using the eigenvalue decomposition of a data covariance (or correlation) matrix or singular value decomposition of a data matrix after normalization of the original data (Armenise, Redmile‐Gordon, Stellacci, Ciccarese, & Rubino, 2013; Everitt & Dunn, 1992). The cumulative contribution rates of the first several PCs > 80% are considered, and the PCA results are usually analyzed according to component and comprehensive scores as Equations 2 and 3 (Abdi & Williams, 2010; Shaw, 2009).

(2) $$Y_{i}=u_{i1}\omega_{1}+u_{i2}\omega_{2}+\ldots +u_{\mathrm{il}}\omega_{l}$$

where, $Y_{i}$ is the component score of the selected PCs, $\omega_{j}$ is the normalized data of factors related to litter amount, *u_ij_* is the factor loading, and *l* is the number of factors.

(3) $$W=\frac{v_{1}Y_{1}+v_{2}Y_{2}+\ldots +v_{m}Y_{m}}{v_{1}+v_{2}+\ldots +v_{m}}$$

where, *W* is the comprehensive score, *v_k_* is the eigenvalue of PCs, and *m* is the number of the selected PCs.

All the descriptive parameters, Pearson correlation analysis, and PCA were obtained using SPSS (version 21.0, International Business Machines Corporation) and R (version 3.3.1, https://www.r-project.org/) for Windows.

### 2.5 Geostatistical analysis

The spatial variability of litter amount was conducted using ArcGIS (version 10.2, http://developers.arcgis.com/). Based on a semivariogram and a cross‐variogram, appropriate models were fitted and the input parameters for the spatial interpolation of kriging and cokriging were provided (Cressie, 1993; Goovaerts, 1997). Three parameters of nugget (C_0_), sill (C + C_0_), and range (A) can show the spatial dependence of litter amount (Burgos, Madejón, Pérez‐de‐Mora, & Cabrera, 2006), and the degree of spatial autocorrelation can be evaluated using structure variance ratio, that is, the value of C_0_/(C_0_ + C) (Jiang et al., 2017).

In a spatial context, the kriging method can use the data in the neighborhood to predict the value of the litter amount at a location where it has not been measured (Wackernagel, 1994). The ordinary kriging estimates were calculated as Equation 4:

(4) $$Z_{\text{OK}}\left(x_{0}\right)=\sum_{i=1}^{a}\lambda_{i}Z\left(x_{i}\right)$$

where, *Z*_OK_(*x*_0_) is the predicted value at the location of *x*_0_; *Z*(*x_i_*) is the measured value at the sampling site *x_i_*, *λ_i_* is a weighting coefficient, which can be calculated according to the unbiasedness, optimality and the Lagrange minimization principle (Sakata, Ashida, & Zako, 2004); and *a* is the number of sites within the search neighborhood around *x*_0_ used for the prediction.

Cokriging is an interpolation method used to combine the litter amount and auxiliary variable while accounting for the spatial pattern and sampling procedure (Goovaerts, 2019). The ordinary cokriging estimates were calculated as Equation 5:

(5) $$Z_{\text{COK}}\left(x_{0}\right)=\sum_{j=1}^{b}\lambda_{1j}Z_{1}(x_{j})+\sum_{k=1}^{c}\lambda_{2k}Z_{2}\left(x_{k}\right)$$

where, *Z*_COK_(*x*_0_) is the predicted value at the location of *x*_0_; *Z*_1_(*x_j_*), target variable, and *Z*_2_(*x_k_*), auxiliary variable, are the measured values at the sampling site *x_j_* and *x_k_*; *λ*_1_ *_j_* and *λ*_2_ *_k_* are weighting coefficients, and *b* and *c* are the number of *Z*_1_(*x_j_*) and *Z*_2_(*x_k_*) within the search neighborhood around *x*_0_ used for the prediction.

### 2.6 Accuracy evaluation

The accuracy of the spatial distribution map was checked using the cross‐validation approach. Five indicators, including mean error (*ME*), mean standardized error (*MSE*), average standard error (*ASE*), root‐mean‐square error (*RMSE*), and root‐mean‐square standardized error (*RMSSE*), were used to evaluate the performance of ordinary kriging and cokriging predictions and calculated as follows (Ali & Othman, 2017; Johnston, Hoef, Krivoruchko, & Lucas, 2001; Zapatarios, Rivero, Naja, & Goovaert, 2012):

(6) $$ME=\frac{\sum_{i=1}^{n}\left(P_{i}-O_{i}\right)}{n}$$

(7) $$MSE=\frac{\sum_{i=1}^{n}\left(P_{i}-O_{i}\right)}{n\sigma \left(O_{i}\right)}$$

(8) $$ASE=\sqrt{\frac{\sum_{i=1}^{n}\sigma {\left(O_{i}\right)}^{2}}{n}}$$

(9) $$RMSE=\sqrt{\frac{\sum_{i=1}^{n}{\left(P_{i}-O_{i}\right)}^{2}}{n}}$$

(10) $$RMSSE=\sqrt{\frac{\sum_{i=1}^{n}{\left[\left(P_{i}-O_{i}\right)/\sigma \left(O_{i}\right)\right]}^{2}}{n}}$$

where, *P_i_* is the measured value, *O_i_* is the predicted value, *σ*(*O_i_*) is the standard error of *O_i_*, and *n* is the number of data pairs. The *ME* provides a bias of predicted value, the *ASE* and *RMSE* provide the accuracy between the predicted value and measured value, and the *MSE* and *RMSSE* provide the accuracy of the standard error.

### 2.7 Prediction procedure

Prediction of the litter amount was carried out following five steps: (1) The original datasets were processed using a normal distribution test and logarithm transformation if necessary; (2) The correlations between the target variable (litter amount) and influencing factors (litter properties and stand characteristics) were tested to select the auxiliary variable *W*_1_ (the explanatory variable with the highest correlation with the target variable); (3) When the PCA was applied based on the results of correlation analysis (*p* < .05) (Camacho & Ferrer, 2014), a comprehensive score was calculated as an auxiliary variable *W*_2_; (4) The ordinary kriging and cokriging (auxiliary variables were *W*_1_ and *W*_2_, respectively) were used to estimate the spatial distribution of the target variable; and (5) The accuracy of the three interpolation methods was compared using cross‐validation.

## 3 RESULTS

### 3.1 Descriptive statistics and normal distribution test

The means and *SD*s of litter amount and litter properties varied across decomposed horizons. The litter amount ranged from 6.83 to 54.77 t/ha with a mean of 19.65 t/ha and an *SD* of 8.36 t/ha in the F horizon, and it ranged from 3.86 to 24.86 t/ha with a mean of 10.37 t/ha and an *SD* of 4.37 t/ha in the H horizon. The F horizon had higher mean concentrations of OC (453.22 g/kg), TN (21.77 g/kg), OC:TN (21.31), OC:TP (416.80), and OC:TP (19.93) than the H horizon for all sampling points. The litter amount data did not follow a normal distribution and had skewness and kurtosis values larger than 0.5. Litter amounts in the F and H horizons were both logarithmically transformed to pass the K–S statistical test (Table 1).

**Table 1:** Descriptive statistics of litter amount and litter properties (*n* = 100)

Item	Horizon	Mean	Max.	Min.	*SD*	CV (%)	Skewness	Kurtosis	K‐S
Litter amount (t/ha)	F	19.65	54.77	6.83	8.36	42.70	1.03	2.08	0.08
	H	10.37	24.86	3.86	4.37	42.14	1.05	1.09	0.10
Moisture content (%)	F	64.05	99.27	34.29	15.39	24.03	−0.73	0.85	0.97
	H	178.58	299.06	84.47	42.15	23.60	0.19	0.38	0.83
OC (g/kg)	F	453.22	883.41	256.30	93.73	20.68	1.89	7.24	0.16
	H	358.67	563.72	104.92	108.26	30.18	0.08	−0.55	0.84
TN (g/kg)	F	21.77	32.34	14.20	3.01	13.83	−0.25	0.94	0.10
	H	19.28	25.74	11.74	2.67	13.85	−0.31	0.11	0.82
TP (g/kg)	F	1.12	2.35	0.57	0.22	19.64	1.77	8.56	0.13
	H	3.18	5.77	1.35	0.82	25.79	0.44	−0.05	0.71
OC:TN	F	21.31	49.42	11.90	6.07	28.48	2.28	7.18	0.01
	H	18.99	45.19	5.79	6.78	35.70	0.89	1.69	0.31
OC:TP	F	416.80	777.24	182.98	110.01	26.39	0.95	1.58	0.06
	H	120.82	366.91	39.66	53.98	44.68	1.53	4.30	0.27
TN:TP	F	19.93	35.01	9.27	4.02	20.17	0.69	1.99	0.25
	H	6.48	13.58	3.05	1.95	30.09	0.61	0.61	0.47

Abbreviations: CV, coefficient of variation; F, semidecomposed horizon; H, complete decomposed horizon; K‐S, significance level of Kolmogorov–Smirnov test for normality; OC, organic carbon; *SD*, standard deviation; TN, total nitrogen; TP, total phosphorous.

The coefficient of variation (CV) is the strongest index to distinguish variability. The CVs of litter amount and moisture content in the F horizon were 0.56% and 0.43% higher than those in the H horizon, respectively. However, the CV of litter OC, TN, TP, OC:TN, OC:TP, and OC:TP concentrations exhibited a reverse trend of being lower by 9.50%, 0.02%, 6.15%, 7.22%, 18.29%, and 9.92%, respectively, in the F horizon than in the H horizon. The CVs of stand characteristics were within the range of 6.17%–56.72%, indicating a small or moderate spatial discretization of stand characteristics in natural spruce–fir mixed forest (Table 2).

**Table 2:** Descriptive statistics of stand characteristics (*n* = 100)

Item	Mean	Max.	Min.	*SD*	CV (%)	Skewness	Kurtosis	K‐S
Canopy density	0.81	0.94	0.62	0.05	6.17	−0.99	1.83	0.19
Species number (stem/ha)	500	800	200	200	34.35	0.15	−0.43	0.01
Stem number (stem/ha)	1,100	4,700	200	600	56.72	2.08	8.54	0.09
Gleason index (*D*)	0.99	1.74	0.43	0.34	34.34	0.15	−0.43	0.01
Shannon Wiener index (*H′*)	1.26	1.97	0.33	0.39	30.95	−0.44	−0.36	0.48
Pielous index (*J)*	0.86	1.00	0.34	0.12	13.95	−1.72	4.08	0.02
Proportion of conifer species (%)	44.7	100.0	0.0	18.0	46.9	0.29	0.19	0.13
Proportion of conifer stems (%)	52.1	100.0	0.0	23.5	54.7	−0.09	−1.09	0.59
DBH (cm)	15.7	26.6	8.6	3.2	20.2	0.60	0.89	0.50
Basal area (m^2^/ha)	2.0	5.6	5.8	0.8	41.5	1.33	3.03	0.13

Abbreviations: CV, coefficient of variation; DBH, diameter at breast height; K‐S, significance level of Kolmogorov–Smirnov test for normality; *SD*, standard deviation.

### 3.2 Selection and processing of auxiliary variables

#### 3.2.1 Pearson correlation analysis

**Figure 2:**
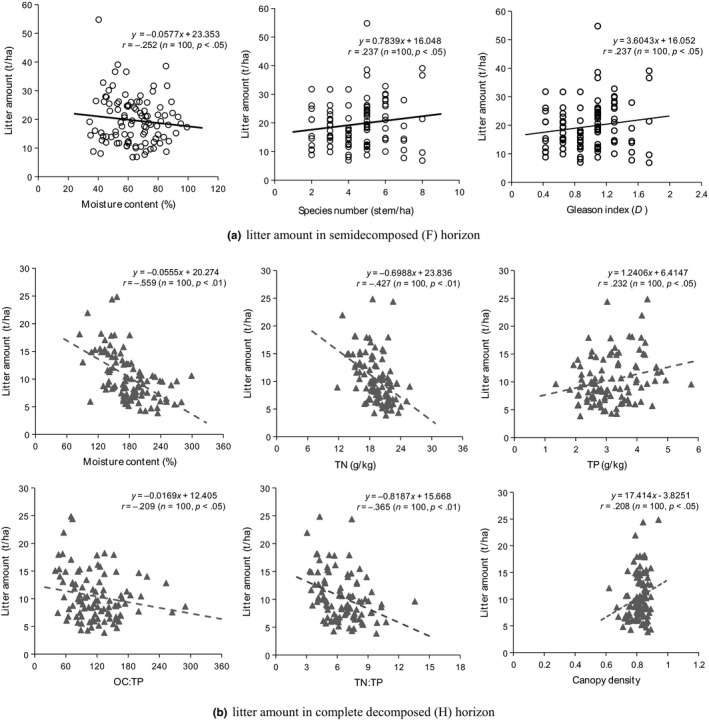
Correlation of litter amount with litter properties and stand characteristics. The numeric data included in the graphs represent the following: linear equation (*y* = *fx* + *g*), correlation coefficient (*r*), the number of samples (*n*), and test of significance (*p*)

The correlation analysis exhibited some differences between the two decomposed horizons (Figure 2). In the F horizon, litter amount had positive correlations with tree species number (*r* = .237, *p* < .05) and Gleason index (*r* = .237, *p* < .05), but it had a negative correlation with moisture content (*r* = −.252, *p* < .05). In the H horizon, litter amount showed a significant relationship with moisture content (*r* = −.559, *p* < .01), TN (*r* = −.427, *p* < .01), TP (*r* = .232, *p* < .05), OC:TP (*r* = −.209, *p* < .05), TN:TP (*r* = −.365, *p* < .01), and canopy density (*r* = .208, *p* < .05), and the strongest relationship was found between litter amount and moisture content. It is likely that litter with a higher moisture content was not beneficial to increasing the litter amount. The proportion of conifer stems and species had similar effects on litter amount in the two decomposed horizons. Nevertheless, the correlation of litter amount with OC was rather weak, as were correlations with DBH and basal area.

#### 3.2.2 Principal component analysis

Litter moisture content, TN, TP, OC:TP, TN:TP, canopy density, species number and Gleason index may be affected by the same regionalized phenomenon or spatial process with litter amount in the study area, so they can be deemed to be a cooperative regionalized variable. The total variance explained by moisture content, species number and Gleason index in the F horizon, and moisture content, TN, TP, OC:TP, TN:TP, canopy density in the H horizon, are shown in Table 3. It was seen that the first two PCs in the F horizon explained 66.84% and 33.15% of the variance, collectively explaining 99.98% of the total variance. The first three PCs in the H horizon explained 84.25% of the total variance among six variables, where the first component (PC1) contributed 45.08%, the second component (PC2) contributed 22.81%, and the third (PC3) 16.35% of the total variance. That is, the first two PCs in the F horizon and the first three PCs in the H horizon have summarized most information of stand characteristics and litter properties.

**Table 3:** Total variance explained by principal components (*n* = 100)

Horizon	Component	Initial eigenvalue			Extraction of square and load		
		Total	Variance (%)	Cumulation (%)	Total	Variance (%)	Cumulation (%)
F	PC1	1.416	66.84	66.84	1.416	66.84	66.84
	PC2	0.997	33.15	99.98	0.997	33.15	99.98
	PC3	0.005	0.01	100.00			
H	PC1	1.645	45.08	45.08	1.645	45.08	45.08
	PC2	1.170	22.81	67.89	1.170	22.81	67.89
	PC3	0.991	16.35	84.25	0.991	16.35	84.25
	PC4	0.826	11.38	95.63			
	PC5	0.475	3.76	99.39			
	PC6	0.192	0.61	100.00			

Abbreviations: F, semidecomposed horizon; H, complete decomposed horizon; PC, principal component.

The PCA result showed that complex relationships existed between litter amount and environmental factors in the study area (Table 4). In the F horizon, PC1 decreased with the increase of species number (−0.705) and Gleason index (−0.705), and PC2 reflected moisture content (0.997). In the H horizon, PC1 had stronger negative correlations with OC:TP (−0.466) and TN:TP ratios (−0.568), and the increasing positive effect of TP (0.486) on PC1 was shown. The PC2 mainly reflected moisture content (−0.528) and TN concentration (−0.676), and PC3 increased with canopy density (0.970).

**Table 4:** Component score coefficient matrix and comprehensive score calculation (*n* = 100)

Horizon	Variable	PC1	PC2	PC3	Component and comprehensive score
F	Moisture content (F_1_)		0.997		$Y_{F_{1}}=0\times \omega_{F_{1}}-0.705\times \omega_{F_{2}}-0.705\times \omega_{F_{3}}$ $Y_{F_{2}}=0.997\times \omega_{F_{1}}+0\times \omega_{F_{2}}+0\times \omega_{F_{3}}$ $W_{F}=\frac{0.6684\times Y_{F_{1}}+0.3315\times Y_{F_{2}}}{0.6684+0.3315}$
	Species number (F_2_)	−0.705			
	Gleason index (F_3_)	−0.705			
H	Moisture content (H_1_)	−0.364	−0.528		$\begin{array}{c} Y_{H_{1}}=-0.364\times \omega_{H_{1}}-0.267\times \omega_{H_{2}}+0.486\times \omega_{H_{3}}\\ -0.466\times \omega_{H_{4}}-0.568\times \omega_{H_{5}}+0.140\times \omega_{H_{6}} \end{array}$ $\begin{array}{c} Y_{H_{2}}=-0.528\times \omega_{H_{1}}-0.676\times \omega_{H_{2}}-0.428\times \omega_{H_{3}}\\ +0.266\times \omega_{H_{4}}+0\times \omega_{H_{5}}+0\times \omega_{H_{6}} \end{array}$ $\begin{array}{c} Y_{H_{3}}=0\times \omega_{H_{1}}+0.175\times \omega_{H_{2}}+0\times \omega_{H_{3}}\\ +0\times \omega_{H_{4}}+0.151\times \omega_{H_{5}}+0.970\times \omega_{H_{6}} \end{array}$ $W_{H}=\frac{0.4508\times Y_{H_{1}}+0.2281\times Y_{H_{2}}+0.1635\times Y_{H_{3}}}{0.4508+0.2281+0.1635}$
	TN (H_2_)	−0.267	−0.676	0.175	
	TP (H_3_)	0.486	−0.428		
	OC:TP (H_4_)	−0.466	0.266		
	TN:TP (H_5_)	−0.568		0.151	
	Canopy density (H_6_)	0.140		0.970	

Abbreviations: F, semidecomposed horizon; H, complete decomposed horizon; PC, principal component; *W*_F_ and *W*_H_, the comprehensive scores in the F and H horizons, respectively; $Y_{F_{1}}$ and $Y_{F_{2}}$, the values of the first two PCs, respectively; $Y_{H_{1}}$,$Y_{H_{2}}$, and $Y_{H_{3}}$, the values of the first three PCs, respectively; $\omega_{F_{i}}$ (i.e., F_1_, moisture content; F_2_, species number; F_3_, Gleason index), the normalized data of factors related to litter amount in the F horizon; $\omega_{H_{i}}$ (i.e., H_1_, moisture content; H_2_, TN; H_3_, TP; H_4_, OC:TP; H_5_, TN:TP; H_6_, canopy density), the normalized data of factors related to litter amount in the H horizon.

### 3.3 Semivariogram analysis

According to the results of the Pearson correlation analysis and PCA, three spatial interpolation methods including OK, COK_P_ which took the strongest correlation with litter amount (litter moisture content in the F and H horizons) as an auxiliary variable, and COK_PCA_ which took the comprehensive score (*W*_F_ in the F horizon and *W*_H_ in the H horizon) as an auxiliary variable were applied. Based on different interpolation methods, the semivariogram of litter amount in this study area was analyzed with the parameters shown in Table 5. The spherical model was best fitted to the semivariogram as for OK (C_0_/C_0_ + C = 43.7%), COK_P_ (C_0_/C_0_ + C = 30.3%), and COK_PCA_ (C_0_/C_0_ + C = 26.0%) in the F horizon, while OK, COK_P_, and COK_PCA_ were all fitted with an exponential model in the H horizon, with structure variance ratios of 41.8%, 26.4%, and 25.5%, respectively. The structure variance ratios of litter amount were less than 75%, revealing a medium spatial autocorrelation, with a range between 18.8 m and 53.6 m.

**Table 5:** Parameters of semivariogram analysis for litter amount (*n* = 100)

Horizon	Interpolation method	Variable	Model	Nugget (C_0_)	Sill (C_0_ + C)	Range (A, m)	Structure variance ratio (C_0_/C_0_ + C, %)
F	OK	Litter amount	Spherical	0.080	0.183	18.8	43.7
	COK_P_	Cov (litter amount, moisture content)	Spherical	0.056	0.185	19.4	30.3
	COK_PCA_	Cov (litter amount, *W*_F_)	Spherical	0.051	0.196	18.8	26.0
H	OK	Litter amount	Exponential	0.079	0.189	53.6	41.8
	COK_P_	Cov (litter amount, moisture content)	Exponential	0.046	0.176	26.5	26.4
	COK_PCA_	Cov (litter amount, *W*_H_)	Exponential	0.037	0.145	33.8	25.5

Abbreviations: COK_P_, ordinary cokriging with litter moisture content as an auxiliary variable; COK_PCA_, ordinary cokriging with comprehensive score as an auxiliary variable; F, semidecomposed horizon; H, complete decomposed horizon; OK, ordinary kriging; *W*_F_ and *W*_H_, the comprehensive scores in the F and H horizons, respectively.

### 3.4 Spatial distribution pattern

**Figure 3:**
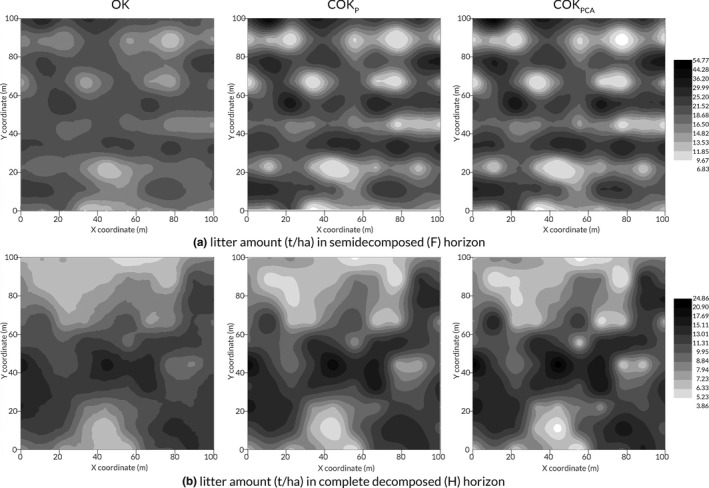
Distribution of litter amount based on the three spatial interpolation methods (*n* = 100). OK, ordinary kriging; COK_P_, ordinary cokriging with litter moisture content as an auxiliary variable; COK_PCA_, ordinary cokriging with the comprehensive score as an auxiliary variable

Based on the semivariogram concept and models, OK, COK_P_, and COK_PCA_ interpolation methods were evaluated for the spatial distribution of litter amount in the two decomposed horizons (Figure 3). Litter amount in the study area exhibited a patchy and heterogeneous distribution for both horizons. In the F horizon, litter amount showed several high‐value centers but was discrete ranging between 29.99 and 54.77 t/ha. The spatial distribution differed along the south–north direction, but not for the west–east direction. In the H horizon, litter amount in the northwestern part had lower values of 3.86–7.94 t/ha, and they were mainly concentrated on the middle and southeastern aspect in the range of 11.31–24.86 t/ha. In both horizons, the spatial prediction map of litter amount using COK_P_ and COK_PCA_ showed that the distribution trend of litter amount was similar to that estimated using OK, but it was less spatially detailed or more uniform. Furthermore, COK_PCA_ interpolation had the most spatial heterogeneity, revealing the randomness and dependence of litter amount spatial distribution in certain local areas.

### 3.5 Estimation accuracy comparison

The estimation accuracy was listed for OK, COK_P_, and COK_PCA_ to emphasize differences among the three datasets (Table 6). An *ME* close to zero indicates a lack of bias, and an *MSE* close to zero and an *RMSSE* close to one indicate smaller estimation errors, while the *ASE* and *RMSE* should be as small as possible. In the F horizon, where minimum *ASE* was 8.681, higher accuracy between the predicted value and the measured value was found using COK_PCA_. The *RMSE* decreased from 9.719 using OK to 9.439 and 9.200 using COK_P_ and COK_PCA_, respectively. For COK_PCA_, the *ME* and *MSE* were close to zero and *RMSSE* was close to one compared with the OK and COK_P_ datasets. The five indicators also showed better predictions using COK_PCA_ for the H horizon. In both horizons, the estimation accuracy showed COK_PCA_ > COK_P_>OK.

**Table 6:** Accuracy comparison of prediction (*n* = 100)

Horizon	Interpolation method	Variable	*ME*	*MSE*	*ASE*	*RMSE*	*RMSSE*
F	OK	Litter amount	−0.226	−0.112	8.818	9.719	1.155
	COK_P_	Cov (litter amount, moisture content)	−0.169	−0.100	8.802	9.439	1.138
	COK_PCA_	Cov (litter amount, *W*_F_)	−0.078	−0.084	8.681	9.200	1.105
H	OK	Litter amount	0.095	−0.034	4.129	4.048	0.818
	COK_P_	Cov (litter amount, moisture content)	0.071	0.017	4.033	3.744	0.895
	COK_PCA_	Cov (litter amount, *W*_H_)	0.051	0.005	3.776	3.571	0.959

Abbreviations: *ASE*, average standard error; COK_P_, ordinary cokriging with litter moisture content as an auxiliary variable; COK_PCA_, ordinary cokriging with comprehensive score as an auxiliary variable; F, semidecomposed horizon; H, complete decomposed horizon; *ME*, mean error; *MSE*, mean standardized error; OK, ordinary kriging; *RMSE*, root‐mean‐square error; *RMSSE*, root‐mean‐square standardized error; *W*_F_ and *W*_H_, the comprehensive scores in the F and H horizons, respectively.

## 4 DISCUSSION

We collected 100 litter samples from a 1‐ha plot for each decomposed horizon. Litter amount varied with the degree of decomposition, and it was 19.65 t/ha in the F horizon and 10.37 t/ha in the H horizon. The average total litter amount was 30.02 t/ha, which is much higher than the mean (4.66 t/ha) reported by Wen and He (2016) for the litter amount of 1,864 sampling sites collected from 2000 to 2014 in China. The mean litter amount in natural forests of three coniferous species (*Pinus pinaster*, *Abies borisii‐regis,* and *Pinus nigra*) and one deciduous species (*Fagus silvatica*) in northern Greece (Kavvadias, Alifragis, Tsiontsis, Brofas, & Stamatelos, 2001) is lower than our data. This could be attributed to the fact that spruce and fir with higher fiber and secondary metabolite concentrations are not easily decomposed (Hendricks & Boring, 1992; Monk & Gabrieison, 1985). The litter amount in our study was higher than that in similar spruce–fir mixed forest in Liaoning Province, China (13.52 t/ha) reported by Liu et al. (2017). The study of spatial heterogeneity requires a higher density of sampling points (100 subplots per hectare). However, some studies have been conducted with fewer points or have been carried out at larger scales to calculate average values instead. Moreover, it is of great importance to focus on uniform standards for research methods.

The factors of stand characteristics and litter properties that were related to litter amount varied between the F and H horizons. The litter amount in the F horizon was significantly related to moisture content, species number, and Gleason index. This suggests that litter quality may be different between tree species (Staelens et al., 2011). We found that litter amount in the H horizon was significantly correlated with moisture content, TN, TP, OC:TP, TN:TP, and canopy density. The litter TN, TP, and TN:TP can affect the rate of litter decay and the leaching and release of N (Berg & McClaugherty, 2003). However, the finding that litter amount in the H horizon was significantly and negatively related to TN is in contrast with Scott and Binkley (1997). A positive or negative effect of site quality on litter amount in the F and H horizons beneath different tree species was noticed by Kavvadias et al. (2001). Canopy structure was likely to influence the important factors affecting the litter decomposition processes, such as the temperature and moisture content of the forest floor (Sariyildiz, 2008). Therefore, canopy density may affect the spatial variability of litter amount. Our results indicated that litter amount had the highest correlation with litter moisture content in both horizons. Previous studies also found effects of litter moisture content on litter decomposition and animal activities (Halupa & Howes, 1995; Kavvadias et al., 2001; Levings & Windsor, 1984). Traditionally, much slower litter decomposition rates and fewer groups of arthropods in these moisture‐limited forests resulted in litter accumulation.

The PCA summarized the relationships between litter amount and multiple factors. In our study, two PCs in the F horizon and three PCs in the H horizon were selected explaining more than 80% of total variance. In the F horizon, factor loadings (absolute values) were higher for stand characteristics (species number and Gleason index) in PC1, which was considered the *stand component*. The PC2 showed large positive loadings for litter moisture content and was considered the *litter component*. In the H horizon, litter properties in PC1 (TP, OC:TP and TN:TP) and PC2 (moisture content and TN) with the highest factor loading were considered *litter components*, and PC3 represented an individual contribution from canopy density and was considered the *stand component*. Obviously, stand characteristics had a higher contribution rate than litter properties in the F horizon, but an opposite result was found in the H horizon. The different processes and effects in each horizon suggested that stand characteristics and litter properties mainly act on the F and H horizon, respectively (Andrews, Karlen, & Mitchell, 2002; Askari & Holden, 2015; Govaerts, Sayre, & Deckers, 2006). Before the peak of fall, litter in the F horizon was exposed at the soil surface, which is highly influenced by the light, precipitation, structure, and species composition of forests. On account of the H horizon beneath the F horizon, stand characteristics had relatively weaker effects on litter in this horizon.

According to the fitted spherical or exponential models, the spatial structure variance ratio in the H horizon was smaller than the F horizon, revealing that it had a stronger spatial autocorrelation. The spatial variability of litter amount may be influenced by many factors. Usually, a strong spatial autocorrelation of litter amount can be attributed to intrinsic factors, and a weak spatial autocorrelation can be attributed to extrinsic factors (Chang, Scrimshaw, Emmerson, & Lester, 1998). The variation range was from 18.8 to 19.4 m in the F horizon and 26.5 to 53.59 m in the H horizon, with both ranges being greater than 10 m. Therefore, the sampling distance set in this study was appropriate. As shown, the spatial pattern in litter amount varied across horizons. Litter amount in the H horizon demonstrated a more complex spatial distribution than in the F horizon, implying that the H horizon may be easily affected by environmental factors. In the F horizon, litter amount showed several high‐value centers and varied along the north–south direction. In the H horizon, lower litter amount was found on the northwestern aspect and higher litter amount was mainly concentrated in the middle and southeastern part. This may also be related to the spatial distribution of tree species and size in addition to the factors previously mentioned in our study. For example, Xia et al. (2015) noticed that different DBH of trees may result in differences in litter input and that the pattern of trees with larger DBH may play a more important role. The spatial patterns of litter amount imply that complex material dynamics exist in forest ecosystems.

In the F and H horizons, the estimated spatial distribution of litter amount using OK, COK_P_, and COK_PCA_ methods was similar to each other but had different spatial details. Both COK_P_ and COK_PCA_ showed an improvement in the prediction of litter amount in comparison to OK. The COK prediction presented lower bias (*ME*), smaller prediction errors (*MSE* and *RMSSE*), and higher accuracy (*ASE* and *RMSE*), simultaneously. Our results agree with Chang et al. (1998) and Yang et al. (2016), who also found that cokriging was superior to kriging. This is mainly because the OK interpolation only considered the spatial information of litter amount, but the COK interpolation improved the estimation accuracy of litter amount by using an auxiliary variable. In previous studies, the spatial factor that had the strongest correlation with the target variable was taken as the auxiliary variable, and to some extent, the influence of other spatial factors on the target variable was ignored (Basaran et al., 2011). In the present study, the litter moisture content, which had the strongest correlation with litter amount, and the comprehensive scores based on PCA in the F and H horizons were used as auxiliary variables to carry out the COK interpolation, respectively. The degree of improvement using COK_PCA_ prediction was obviously higher than that using COK_P_, revealing that COK_PCA_ can reduce the loss of raw data and information, simplify the data structure and avoid subjective randomness to accurately predict and simulate the spatial pattern of litter amount in the study site.

## 5 CONCLUSIONS

This paper presented the first application and comparison of OK, COK_P_, and COK_PCA_ to study the spatial distribution of litter amount in the F and H horizons at the local scale (1‐ha) from a natural mixed forest stand. Our results indicated that litter amount varied with horizons, with a range of 6.83–54.77 t/ha in the F horizon and 3.86–24.86 t/ha in the H horizon. In both horizons, litter amount had the strongest negative correlation with litter moisture content. Spatial autocorrelation of litter amount differed in the F and H horizons, but with a clear patchy and heterogeneous pattern. The cokriging, which used litter moisture content and comprehensive scores as auxiliary variables to predict the litter amount, generated a better map than the kriging. The application of COK_PCA_ led to a more unbiased predicted value (*ME* closer to zero), more accuracy between the predicted value and measured value (smaller *ASE* and *RMSE*) and smaller prediction errors (*MSE* closer to zero and *RMSSE* closer to one) compared with the OK and COK_P_ datasets. The combination of Pearson correlation analysis especially PCA with geostatistics offered brief and concise information about the spatial variability of litter amount and its relationship with stand characteristics and litter properties at a small scale. The feasibility and accuracy of using cokriging based on principal component analysis not only provided a scientific interpolation method for predicting the spatial distribution of litter amount but also valuable information for better enriching and developing geostatistics in forest ecosystems.

## CONFLICT OF INTEREST

We declared that we have no conflict of interest.

## AUTHOR CONTRIBUTIONS

Qianqian QIN involved in conceptualization, methodology, software, formal analysis, writing original draft, and supervision. Qianqian QIN and Haiyan WANG performed validation and visualization. Qianqian QIN, Xiang LI, Yalin XIE, and Yonglin ZHENG investigated the data. Haiyan WANG involved in data curation, project administration, and funding acquisition. Haiyan WANG and Xiangdong LEI involved in resources. Qianqian QIN, Haiyan WANG, and Xiangdong LEI have written, reviewed, and edited the article.

## Data Availability

All data generated or analyzed during this study are included in this published article.
